# MicroRNA Profiling Identifies Age-Associated MicroRNAs and Potential Biomarkers for Early Diagnosis of Autism

**DOI:** 10.3390/ijms26052044

**Published:** 2025-02-26

**Authors:** Salam Salloum-Asfar, Samia M. Ltaief, Rowaida Z. Taha, Wared Nour-Eldine, Sara A. Abdulla, Abeer R. Al-Shammari

**Affiliations:** 1 Neurological Disorders Research Center, Qatar Biomedical Research Institute, Hamad Bin Khalifa University, Qatar Foundation, Doha P.O. Box 34110, Qatar; ssalloumasfar@hbku.edu.qa (S.S.-A.); sltaief@hbku.edu.qa (S.M.L.); rotaha@hbku.edu.qa (R.Z.T.); wnoureldine@hbku.edu.qa (W.N.-E.); 2 College of Health and Life Sciences, Hamad Bin Khalifa University, Qatar Foundation, Doha P.O. Box 34110, Qatar

**Keywords:** ASD, autism, miRNAs, plasma, diagnosis, biomarkers, logistic regression

## Abstract

Autism spectrum disorder (ASD) is a neurodevelopmental disorder in which early diagnosis is critical for effective intervention and improved outcomes. MicroRNAs (miRNAs) are small non-coding RNAs that regulate gene expression and have emerged as promising biomarkers for neurological disorders, including ASD. In our previous discovery study, we identified dysregulated expression of several miRNAs in the plasma samples of children with ASD aged 5–12 years. In this study, we aimed to validate these findings in a younger cohort with ASD (aged 2–4 years) and assess their potential use as biomarkers for the early diagnosis of ASD. A total of 108 young children aged 2–4 years were recruited, including 66 children with ASD and 42 age- and sex-matched controls. Using next-generation sequencing and advanced bioinformatics, we validated the differential expression of 17 miRNAs in ASD, which showed consistent dysregulation across both the current and previous cohorts. We also observed significant correlations between several miRNAs and participants’ age, suggesting that age is a key factor influencing dynamic miRNA changes, particularly in the ASD group. Pathway analysis linked these miRNAs to critical regulatory networks involved in neurodevelopment and immune responses. Finally, we found that a combination of four miRNAs (miR-4433b-5p, miR-15a-5p, miR-335-5p, and miR-1180-3p) exhibited high diagnostic accuracy, with an area under the curve (ROC-AUC) of 0.936 (95% CI = 0.892, 0.980; *p* < 0.001). These findings support the use of this four-miRNA panel as a robust biomarker for early ASD diagnosis and lay the groundwork for future research into miRNA-based diagnostic tools and therapeutic strategies for ASD.

## 1. Introduction

Autism spectrum disorder (ASD) is a multifaceted neurodevelopmental condition that manifests through a diverse array of behavioral and cognitive profiles, challenging our understanding of the human brain [1]. Characterized by difficulties in social communication, restricted interests, and repetitive behaviors, ASD affects approximately 1 in 100 children worldwide [2]; however, its underlying causes and mechanisms remain only partially understood. Currently, ASD is diagnosed based on clinical observation of behavioral symptoms, which risks late or misdiagnosis and, consequently, ineffective therapeutic interventions [3]. Early diagnosis can facilitate timely intervention, improve the quality of life for children with ASD and their parents and reduce the socioeconomic burden [4]. Hence, the emergence of biomarker discovery offers the potential to transform ASD diagnosis from a traditional, phenotypic assessment to an objective and precise diagnosis. The pursuit of reliable biomarkers has encouraged investigators to publish reports on a broad spectrum of molecular biomarkers, including inflammatory cytokines, microparticles, exosomes, autoantibodies, noncoding RNAs, and others [5]. Yet, no approved biomarker for ASD diagnosis is currently available, emphasizing the need for further investigation.

MicroRNAs (miRNAs) constitute a class of short noncoding RNAs, typically 20–24 nucleotides in length, that act as post-transcriptional regulators by either degrading or suppressing the translation of their target messenger RNAs (mRNAs) [6]. A single miRNA can bind to hundreds of mRNAs and thus regulate multiple molecular pathways, highlighting the complexity of miRNA-mediated gene regulation [7]. miRNAs are expressed in the brain in a regional and developmental phase-specific expression pattern [8], thus regulating key mRNAs involved in neural development and functions [9,10]. Importantly, recent studies on autopsies from subjects with ASD demonstrated the dysregulation of miRNAs in various brain regions, including the cerebral cortex, frontal cortex, temporal lobe, and amygdala. Consequently, studies on animal models of ASD and induced pluripotent stem cells (iPSCs) derived from subjects with ASD have identified several miRNA candidates for the diagnosis and prognosis of ASD [11]. Interestingly, a recent systematic review summarized the associations between dysregulated miRNA levels and their target genes with different manifestations of ASD, including memory and learning, neuronal maturation, intelligence, social behavior, and immunity [12]. Importantly, miRNAs exhibit unique features that make them suitable biomarkers, such as (1) their stability in body fluids (e.g., blood, saliva, and cerebrospinal fluid), which allows for non-invasive sampling and reliable detection; (2) tissue-specific expression; and (3) accurate and sensitive detection with high-throughput technologies [13,14].

Previous studies have reported dysregulated miRNAs in the serum and saliva, further supporting the potential of circulating miRNAs as biomarkers for ASD [15,16,17]. Moreover, in our previous study, we identified altered expression of several miRNAs in the plasma of children with ASD aged 5–12 years [18]. In this study, we recruited a total of 108 children aged 2–4 years, including 66 with ASD and 42 age- and sex-matched controls, and analyzed the expression profiles of circulating plasma miRNAs in ASD using next-generation sequencing. The aim of this study is to validate dysregulated miRNA patterns in a younger cohort of children with ASD (aged 2–4 years) and assess the potential use of these plasma miRNAs as diagnostic biomarkers for early ASD detection. Additionally, we explored whether age might influence changes in miRNA levels and investigated the functional relevance of these miRNAs to ASD pathophysiology. To the best of our knowledge, this is the first study investigating circulating plasma miRNAs in very young children with ASD, aged 2–4 years, and in a highly homogeneous cohort. Given the challenges in obtaining blood samples from this age group, our study represents a rare and valuable dataset. While previous studies have focused on older children and adolescents, our findings highlight miRNA dysregulation at an early developmental stage, reinforcing their potential as early biomarkers for ASD diagnosis.

## 2. Results

### 2.1. Study Population Characteristics

The study population shows matching demographic characteristics in terms of age, sex, and nationality, as presented in Table 1. Among the 108 patients, 87 (80.6%) were males, and 21 (19.4%) were females. The male-to-female ratio was almost 4.1, with 33/9 in the control group and 54/12 in the ASD group (*p* = 0.678). The median age was 3.46 (3.11–3.75) years for the ASD group and 3.37 (2.93–3.81) years for the control group; therefore, there were no significant differences in age between the two groups (*p* = 0.504). Moreover, the distribution of nationalities did not show a significant difference between the ASD and control groups (*p* = 0.121, Table 1). The percentages of participants from various nationalities were somewhat comparable: Egyptian (21.2% in ASD vs. 28.6% in control), Qatari (24.2% vs. 11.9%), Syrian (12.1% vs. 26.2%), Yemeni (18.2% vs. 9.5%), Sudanese (9.1% vs. 16.7%), Jordanian (1.5% vs. 2.4%), and other nationalities (13.6% vs. 4.8%). Additionally, there were no differences between children with ASD and controls in terms of consanguinity (*p* = 0.353), method of reproduction (i.e., natural or assisted) (*p* = 0.647), maternal complications (i.e., diabetes, asthma, allergy or hypertension) (*p* = 0.820), pregnancy duration (*p* = 0.401), maternal age at labor (*p* = 0.570), type of delivery (i.e., normal, c-section, or induced) (*p* = 0.254), postnatal complications (i.e., hypoxia or jaundice) (*p* = 0.094) or birth weight (*p* = 0.262).

### 2.2. Differential Expression of Plasma miRNAs in Children with ASD

**Figure 1:**
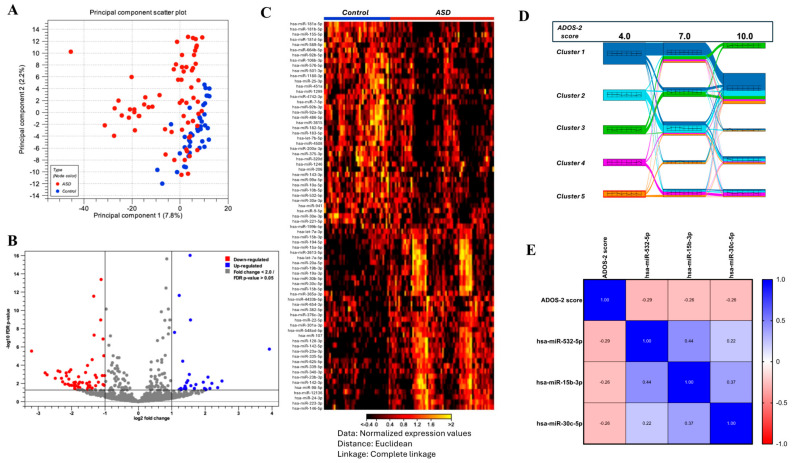
Differential expression of circulating miRNAs in ASD and their associations with ASD severity. (**A**) Principal Component Analysis (PCA) scatter plot demonstrating the separation between the ASD and control groups. (**B**) Volcano plot illustrating the differential expression of miRNAs between the ASD and control groups with a cutoff absolute fold change of 1.5 and FDR adjusted *p*-value < 0.05. (**C**) Heatmap showing miRNA expression levels across individual samples from the ASD and control groups. Each row represents a specific miRNA, and each column corresponds to a sample. The color gradient from black (low expression) to yellow (high expression) reflects relative miRNA expression. (**E**) K-medoids clustering analysis identified five distinct clusters of miRNAs associated with varying levels of ADOS-2 scores, revealing unique expression patterns within each cluster. (**D**) Correlation matrix showing the relationships between selected miRNAs and ASD severity.

We identified 71 miRNAs that were significantly different between the ASD and control groups (Figure 1 and Table S1). Analysis of miRNA expression profiles in children with ASD revealed distinct patterns indicative of the potential role of specific miRNAs in the disorder. A clear separation between the ASD and control samples was observed along the first principal component, which explained 7.8% of the variance, with severe ASD cases clustering separately from controls (Figure 1A). The differential expression of various miRNAs across samples was further delineated with specific miRNAs, such as hsa-miR-181a-5p and hsa-miR-155-5p, which were consistently upregulated in the ASD samples compared with the control samples (Figure 1B). Moreover, the statistical overview provided by the volcano plot highlights miRNAs with significant changes in expression levels, with hsa-miR-3135b markedly upregulated and hsa-miR-4755-3p downregulated in ASD samples. These findings, characterized by a cutoff absolute fold change of 1.5 and FDR adjusted *p*-values below 0.05, indicate that these differentially expressed miRNAs may serve as potential biomarkers for ASD (Figure 1C).

### 2.3. Correlation of Several miRNAs with the Clinical Severity of ASD

We then conducted k-medoids analysis to explore whether the 71 differentially expressed miRNAs were associated with variable degrees of ASD severity. This analysis approach revealed five distinct clusters, in which the distance between miRNAs within a given cluster was small, whereas the distance between independent clusters was large. Interestingly, these five clusters showed associations with ADOS-2 scores of 4, 7 and 10, which corresponded to increasing levels of ASD severity, respectively (Figure 1D). In particular, cluster 1 showed a strong connection to higher ADOS-2 scores of 10, indicating that miRNAs within this cluster were likely associated with more severe symptoms of ASD (Figure 1D). Conversely, cluster 3 was linked to lower ADOS-2 scores of 4, suggesting that miRNAs in this cluster may be involved in milder forms of ASD (Figure 1D). These findings suggest that different miRNA clusters are implicated at varying levels of ASD severity, supporting a potential use of specific miRNAs as biomarkers for stratifying ASD by severity. The list of specific miRNAs within each cluster is included in Table S2.

Furthermore, we conducted a correlation analysis between each miRNA marker and ADOS-2 scores to assess the relationship between individual miRNA expression and ASD severity (Figure 1E). We identified a significant negative correlation between specific miRNAs and ASD severity. For example, hsa-miR-532-5p was negatively correlated with ADOS-2 scores (r = −0.289, *p* = 0.019), suggesting that lower levels of this miRNA are associated with more severe ASD symptoms. Similarly, hsa-miR-15b-3p (r = −0.256, *p* = 0.039) and hsa-miR-30c-5p (r = −0.265, *p* = 0.032) also showed negative correlations with ASD severity (Figure 1E). These findings further reinforce the potential of these miRNAs to serve as biomarkers for assessing the severity of ASD and contribute to our understanding of the pathophysiological mechanisms underlying this disorder.

### 2.4. Correlation of Circulating miRNAs with the Age of Children with ASD

We found significant correlations between several miRNAs and the age of the study participants, mainly in the ASD group, but not in the control group, in both cohorts (Table 2). In particular, nine and thirteen miRNAs were associated with the age of children with ASD at 2–4 years and 5–12 years, respectively (Table 2). However, only three miRNAs correlated with the control group’s age at 2–4 years, and one miRNA was associated with the age of the control group at 5–12 years (Table 2). Among these miRNAs, four miRNAs were shared between the study cohorts, namely hsa-miR-182-5p, hsa-miR-183-5p, hsa-miR-335-5p, and hsa-let-7b-5p, while two additional miRNAs, namely hsa-miR-7-5p and hsa-miR-4742-3p, were within the 17-miRNA signature identified in this study (Table 2). Interestingly, most of the miRNAs were positively correlated with the age of the study cohort at 2–4 years but negatively correlated with the cohort aged 5–12 years. These results indicate that age is a significant factor associated with dynamic changes in miRNAs, specifically in the ASD group.

### 2.5. Validation of the 17-miRNA Signature in Plasma Samples of Children with ASD

In our previous study [18], we reported several altered miRNAs in ASD, which was based on an independent study cohort from a similar population, using a similar analytical approach to that used in this study. Remarkably, we found that 17 miRNAs were common between the two study cohorts (Table 3 and Table S3). Our validated 17-miRNA signature includes hsa-miR-99a-5p, hsa-miR-4433b-5p, hsa-miR-199b-5p, hsa-miR-12136, hsa-miR-15a-5p, hsa-miR-183-5p, hsa-miR-335-5p, hsa-miR-127-3p, hsa-miR-4742-3p, hsa-let-7b-5p, hsa-miR-7-5p, hsa-miR-19a-3p, hsa-miR-3613-5p, hsa-miR-1301-3p, hsa-miR-1180-3p, hsa-miR-182-5p, and hsa-miR-375-3p (Table 3). Interestingly, these 17 miRNAs are involved in a wide range of biological processes, including cell cycle regulation, apoptosis, immune response, and neural development (Table 3). We then performed BSCE analysis and identified dysregulated expression of the target genes of the 17 miRNAs in both blood and brain tissues in independent studies on ASD. These results suggest potential roles for the 17-miRNAs in both immune and neuronal functions in ASD.

### 2.6. Dysregulated Expression of the Target Genes of 17-miRNA Signature in the Blood and Brain Tissues in ASD

We performed BSCE analysis to identify the target genes of the 17-miRNA signature and to determine whether these genes were correlated with altered transcriptomic expression in blood and brain tissues from independent studies on ASD. We found that the 17-miRNAs are known to target a total of 6363 genes. We then analyzed the overlap between our dataset and eight independent studies on ASD, which were conducted on human blood samples (four studies) and human brain tissues (four studies). We found 437 common target genes between our dataset and independent study on whole blood samples in ASD in comparison to the control group (Figure S1A and Table S3) [23]. Additionally, we identified a total of 56 and 78 target genes that were common between our dataset and other studies on leukocytes and lymphocytes of individuals with ASD vs. control group, respectively (Figure S1B,C and Table S3) [20,24]. We also found 1658 common target genes between our dataset and independent study on the blood samples of subjects with ASD features compared to the control group (Figure S1D and Table S3) [25]. These results suggest a potential role for the 17-miRNA signature in regulating immune functions in the circulating blood in individuals with ASD.

Meanwhile, we sought to determine whether our list of differentially expressed miRNAs was also correlated with differential expression of the target genes in brain tissues of subjects with ASD. Interestingly, we identified an overlap of 46, 805, and 348 target genes between our dataset and independent studies conducted on brain samples, cerebral cortex, and prefrontal cortex of subjects with ASD compared to their controls (Figure S2 and Table S4) [26,27]. In addition, we found a total of 41, 288, 340, and 1301 targets genes shared between our dataset and other studies on cerebellar vermis, superior temporal gyrus, occipital lobe, and cerebellum of individuals with ASD vs. controls, respectively (Figure S2 and Table S4) [27,28]. We also identified a total of 101 target genes that were common between our dataset and another study on neurons derived from subjects with ASD compared to the control group (Figure S2 and Table S4) [29]. These observations may indicate that the 17-miRNA signature could be involved in the regulation of neuronal functions in ASD.

Furthermore, the BSCE platform was used to conduct tissue and cell type enrichment analysis to examine the distribution of miRNA targets identified in this study. The analysis revealed significant enrichment in tissues of the nervous system, particularly in the trigeminal ganglion, thalamus, and cerebellum (Table S5). These findings indicate a potential role for the dysregulated miRNAs in neurodevelopmental processes linked to ASD. Additionally, enrichment analysis at the cellular level of the nervous system showed a strong association of miRNA targets with neurons, motor neurons, and astrocytes (Table S5), indicating functional roles of these miRNAs in neuronal functions and communications. Furthermore, immune cells, particularly lymphocytes and B lymphoblastoid cells, were highly enriched, which suggests that immune-related pathways might also be influenced by the identified miRNAs (Table S5). These findings underscore the multi-system regulatory potential of miRNAs in ASD, likely involving both neurodevelopmental and immune system components.

### 2.7. Molecular Functions of the 17-miRNA Signature in ASD

**Figure 2:**
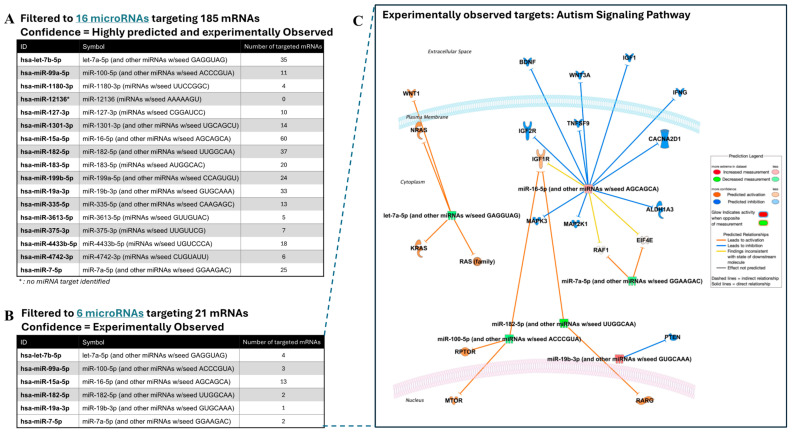
Summary of the targeting activity of 17-miRNA signature and its associated molecular pathways. (**A**) List of 16 highly predicted and experimentally observed miRNAs with their corresponding seed sequences and the number of targeted mRNAs. (**B**) List of 6 experimentally observed miRNAs with their corresponding seed sequences and the number of targeted mRNAs. (**C**) Network diagram of autism signaling pathway illustrating the interactions between these miRNAs and their target genes involved in neurodevelopment and immune responses. Blue lines represent predicted downregulation, while orange lines represent predicted upregulation in the target genes.

We then performed pathway analysis using IPA software to further understand the molecular mechanisms and functions of the dysregulated miRNAs in ASD. We applied two filtering steps. In the first step, we focused on miRNA targets that were both highly predicted and experimentally validated. Among the 17 miRNAs in our dataset, targeting information was available for sixteen miRNAs, while one miRNA, miR-12136, had no identified target, likely due to its classification as a less-studied or novel miRNA. We then applied a second filtering step to focus on pathways linked to Autism Signaling Pathway. After these filtering steps, 16 miRNAs were shown to target a total of 185 mRNAs, revealing two distinct groups of miRNAs: one group of 16 miRNAs that were both highly predicted and experimentally confirmed (Figure 2A) and a second group of 6 miRNAs that had been experimentally observed in previous studies (Figure 2B). Among the 16 miRNAs, miR-15a-5p emerged as key regulator, targeting 60 mRNAs and suggesting its broad regulatory influence in ASD (Figure 2C).

### 2.8. Logistic Regression Model Revealed Predictor Markers for ASD Diagnosis

**Figure 3:**
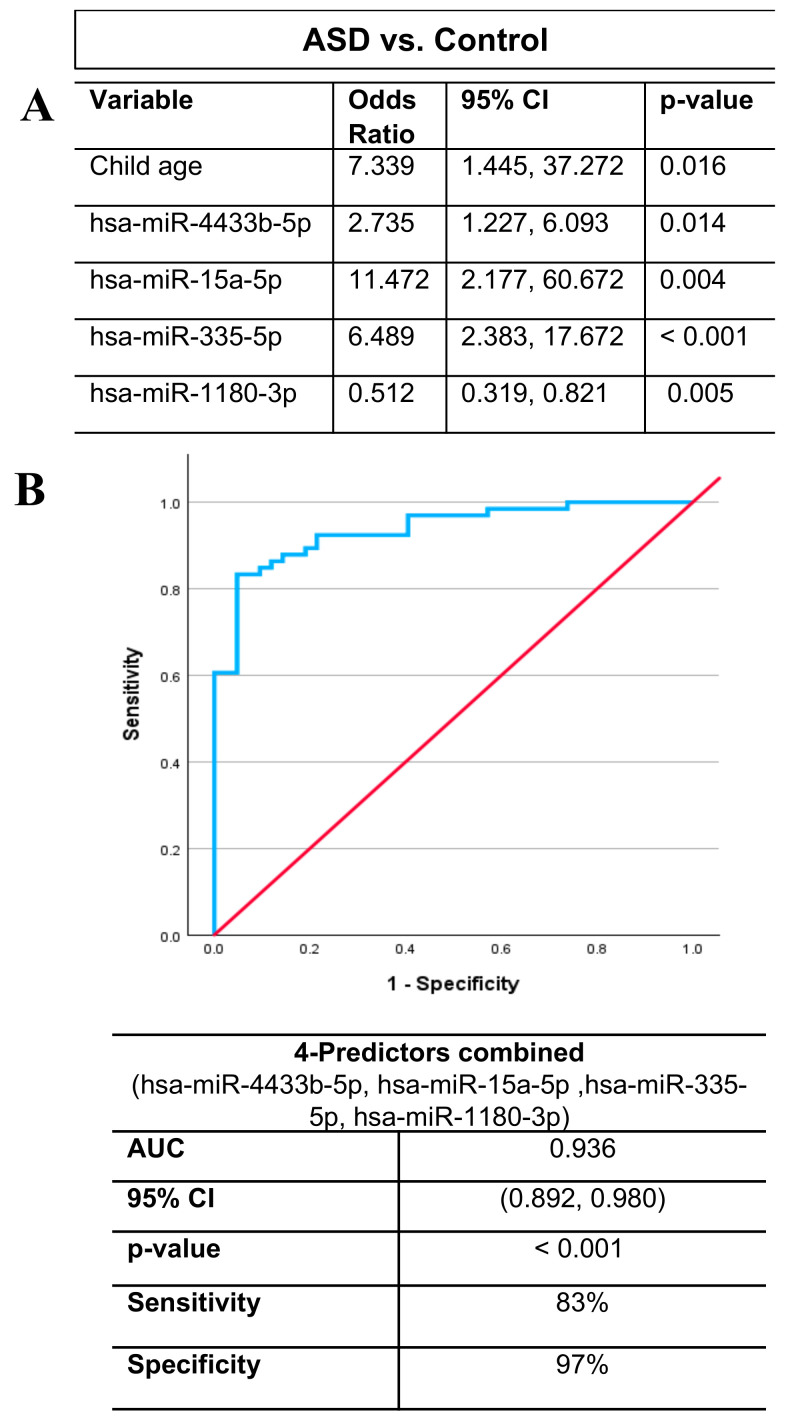
Diagnostic performance of the four selected markers in differentiating between ASD and control cases. (**A**) Binary logistic regression analysis identifies four miRNAs as significant predictors of ASD diagnosis, with the child age retained in the final model. (**B**) Receiver operating characteristic (ROC) curve illustrates the overall diagnostic performance of the combined four-miRNA panel. The area under the curve (AUC), 95% confidence interval (CI), *p*-value, sensitivity, and specificity are displayed in the table for the combined predictors. The blue line represents the ROC curve for the combined predictors, while the red diagonal line represents the reference line.

We performed binary logistic regression analysis to investigate the ability of the significantly altered miRNAs to predict ASD diagnosis. In the initial model, we included all of the 17 differentially expressed miRNAs and we adjusted for the covariates of child age and sex in the model. We found that only four miRNAs and child age remained significant and hence were retained in the final model (Figure 3A). Elevated levels of miRNA-4433b-5p (OR = 2.735; 95% CI = 1.227, 6.093; *p* = 0.014), miRNA-15a-5p (OR = 11.472; 95% CI = 2.177, 60.672; *p* = 0.004), and miRNA-335-5p (OR = 6.489; 95% CI = 2.383, 17.672; *p* < 0.001) were associated with higher odds of having ASD, whereas reduced levels of miRNA-1180-3p (OR = 0.512; 95% CI = 0.319, 0.821; *p* = 0.005) was associated with lower odds of having ASD compared with the control group (Figure 3A).

### 2.9. Diagnostic Accuracy of the Four-miRNA Panel in ASD Detection

We fitted ROC curves to examine the performance of the significant markers that remained in the final logistic regression model (Figure 3B). ROC curve analysis showed that each of the four miRNAs demonstrated significant performance in distinguishing between the ASD and control cases (*p* < 0.01). Specifically, miRNA-4433b-5p showed an area under the curve (AUC) = 0.650 with 95% CI = (0.542, 0.757), miRNA-15a-5p (AUC = 0.723; 95% CI = 0.628, 0.818), miRNA-335-5p (AUC = 0.905; 95% CI = 0.844, 0.965), and miRNA-1180-3p (AUC = 0.228; 95% CI = 0.128, 0.329). An AUC below 0.5 for miRNA-1180-3p indicates lower levels of this miRNA in the ASD group than in the control group. Remarkably, ROC curve analysis demonstrated that the combination of the four miRNAs (miR-4433b-5p, miR-15a-5p, miR-335-5p, and miR-1180-3p) showed the best diagnostic accuracy for ASD (AUC = 0.936; 95% CI = 0.892, 0.980) with high sensitivity and specificity of 83% and 97%, respectively (*p* < 0.001, Figure 3B). These data suggest the potential use of this four-biomarker panel for early ASD diagnosis.

## 3. Discussion

Previously, we discovered altered expression of several miRNAs in the plasma of children with ASD aged 5–12 years [18]. In this study, we aimed to confirm these findings in a younger cohort of ASD subjects aged 2–4 years and evaluate the potential of these plasma miRNAs as diagnostic biomarkers for the early detection of ASD. Notably, we validated the differential expression of 17 miRNAs that showed consistent dysregulation across both our current and previous cohorts. We also investigated whether age affects miRNA levels and explored the functional significance of these miRNAs in relation to ASD pathophysiology. Interestingly, we found significant correlations between several miRNAs and the participants’ age, indicating that age is an important factor involved in regulating miRNA expression, particularly in the ASD group. Pathway analysis connected these validated miRNAs to key regulatory networks involved in neurodevelopment and immune responses. These findings support the potential of the 17 miRNAs as biomarkers for ASD and reveal their functional relevance to ASD pathophysiology. Intriguingly, we found that a combination of four miRNA markers demonstrated strong diagnostic potential with an overall accuracy exceeding 90%. This underscores the utility of these four miRNAs as potential biomarkers for early diagnosis of ASD.

In this study, we validated that 17 miRNAs were consistently dysregulated in both our current and previous cohorts from the same population [18] as well as in other independent studies on ASD [20,21]. Several of these miRNAs, such as hsa-miR-335-5p, hsa-miR-15a-5p, and hsa-miR-183-5p, are well-established in the literature for their roles in brain development and sensory processing, which further supports their relevance to ASD [10,19,20]. Additionally, hsa-miR-7-5p and hsa-let-7b-5p miRNAs are implicated in synaptic plasticity and developmental timing, suggesting their potential involvement in the cognitive and behavioral symptoms observed in ASD [14,22]. Moreover, we found significant correlations between three miRNAs, namely miRNA-532-5p, miR-15b-3p, and miR-30c-5p, and the clinical severity of ASD symptoms, suggesting molecular contributions of these miRNAs to ASD severity. Interestingly, we found that a combination of four miRNAs, namely miR-4433b-5p, miR-15a-5p, miR-335-5p, and miR-1180-3p, demonstrated high diagnostic accuracy for ASD. Previous studies demonstrated that these four miRNAs modulate the expression of various genes involved in neurodevelopment, neural differentiation, and apoptosis [11,19,20,21]. These findings highlight the potential use of these miRNAs as robust biomarkers for ASD and further suggest potential implications of these miRNAs for ASD pathophysiology.

Meanwhile, we sought to determine whether age was a significant factor affecting the levels of circulating miRNAs in individuals with ASD. We investigated the correlation of differentially expressed miRNAs with the age of study participants from two independent cohorts representing different age ranges: 2–4 years and 5–12 years. Remarkably, we found that most of the miRNAs were positively correlated with age in the 2–4-year cohort but negatively correlated with age in the 5–12-year cohort. These results suggest that age is a significant factor associated with dynamic changes in miRNAs, particularly in the ASD group. Currently, there is no evidence for the association of the identified miRNAs with the age of subjects with ASD. However, previous studies have shown a correlation between the expression of hsa-miR-486-5p or hsa-miR-196a-5p and the age of study participants with Parkinson’s disease or glioma conditions, respectively [19,30]. Our findings highlight the dynamic nature of miRNAs with age and their potential contributions to the progression of ASD over time.

We then explored the functional relevance of the 17 validated miRNAs to ASD pathophysiology. BSCE analysis revealed dysregulated expression of the target genes of these miRNAs in both the blood and brain tissues of individuals with ASD [20,23,24,25,26,27,28,29]. For the brain tissues, BSCE analysis showed enrichment of miRNA targets in several brain regions, including trigeminal ganglion, thalamus, and cerebellum, which strongly suggests that these miRNAs are likely involved in neurodevelopmental processes that align with the established understanding of ASD as a neurodevelopmental disorder [8,11]. For example, miR-335-5p and miR-19b-3p, which were upregulated in our study, have been previously implicated in neuronal development and synaptic plasticity, both of which are critical processes disrupted in ASD [10,22]. We found significant enrichment of miRNA targets in neurons, motor neurons, and astrocytes, which further reinforces the role of these miRNAs in neural communications and functions, both of which are frequently disrupted in ASD. In addition to the nervous system, the enrichment of miRNA targets in immune cells, particularly lymphocytes and B lymphoblastoid cells, highlights the potential involvement of immune dysregulation in ASD pathophysiology. This finding is consistent with emerging evidence that links immune responses and inflammation to ASD [19,22]. These observations suggest that our identified miRNAs play multifaceted roles, affecting a range of systems crucial to ASD development. Further investigations into the specific roles of these miRNAs in neuroimmune interactions could provide a deeper understanding of ASD pathogenesis.

Furthermore, we performed IPA analysis and focused on selected pathways known to be linked to the autism signaling pathway. Network analysis demonstrated that these miRNAs interact with several critical genes, including IGF1R, MAPK, and mTOR, which are implicated in key processes such as neuronal growth, synaptic plasticity, and cellular signaling. In particular, three miRNAs—miR-15a-5p, miR-99a-5p, and miR-182-5p—showed significant interactions with genes involved in the IGF1R signaling pathway, which plays a crucial role in brain development. These findings emphasize the important roles of these miRNAs in regulating pathways central to ASD pathophysiology. While our analysis provides strong evidence for their involvement in key regulatory networks, further functional studies are necessary to validate these miRNA–target interactions. Functional studies targeting these miRNAs in relevant cell types and tissues will be critical for confirming their regulatory roles and for exploring their potential as biomarkers or therapeutic targets. To investigate the functional effects of these miRNAs on their targets, we plan to use miRNA mimics and antimiRs in a neuronal cell model to assess their regulatory impact on gene expression. These studies will provide important functional insights into the roles of these miRNAs in ASD-relevant pathways and support their potential translational applications.

Our study has some limitations. The cross-sectional nature of the study limits our ability to establish causality between miRNA expression and ASD progression. Longitudinal studies are essential to assess whether changes in miRNA levels over time correlate with the development of ASD symptoms. Such studies should provide novel insights into the potential of these miRNAs as reliable biomarkers for monitoring disease progression and treatment response. Future research with larger and more diverse cohorts could support the robustness of these miRNAs as diagnostic tools and clarify whether the observed expression patterns remain consistent across different populations and stages of ASD. Additionally, functional studies are also necessary to further investigate how these miRNAs contribute to the molecular underpinnings of ASD. Moreover, the influence of other factors such as environmental exposures, comorbidities, genetic predispositions, and dietary habits on miRNA expression should be explored in future studies to gain a more comprehensive understanding of their roles in ASD. Finally, while our sample size of 108 children (66 ASD and 42 controls) falls within the typical range for pilot studies in biomarker discovery (100–500 subjects) [16,31], larger multi-center validation studies will be necessary to confirm the generalizability and clinical utility of our findings. Moving forward, we aim to validate our four-miRNA panel (miR-4433b-5p, miR-15a-5p, miR-335-5p, and miR-1180-3p) in an independent cohort and apply cross-validation techniques, such as bootstrapping, to further strengthen the robustness of our results.

In conclusion, this study provides evidence supporting the use of miRNAs as biomarkers for ASD. We identified a four-miRNA panel that can distinguish between ASD and control cases with a high diagnostic accuracy exceeding 90%. We found that age is a significant factor associated with altered levels of circulating miRNAs in ASD. Additionally, we determined the functional relevance of these miRNAs to ASD pathophysiology. The findings of this study lay a foundation for future research aimed at developing miRNA-based diagnostic tools and therapeutic strategies for early intervention in ASD. Given the complexity of ASD, future studies should adopt a multifaceted approach that combines miRNA profiling with genetic, environmental, and clinical data to fully elucidate the molecular underpinnings of this disorder.

## 4. Methods

### 4.1. Study Participants

This study included 108 participants, with 66 participants in the ASD group and 42 participants in the control group. All children with ASD were recruited from the Child Development Center in Rumailah Hospital of Hamad Medical Corporation (HMC) in Doha, Qatar, and their matched control participants were recruited from Al-Wajbah Health Center, which is part of the Primary Health Care Corporation (PHCC) in Doha, Qatar. This study was conducted in accordance with the guidelines of the Declaration of Helsinki. Written informed consent was obtained from the families of all study participants before enrollment in this study.

All subjects were initially screened using a questionnaire to ensure they met the study eligibility criteria, as described in our previous publications [32,33]. All enrolled subjects met the following criteria: aged 2–4 years; Arabic ethnicity; born in Qatar; their mothers lived in Qatar during most of their pregnancy; enrolled subjects mostly lived in Qatar since birth; no immune conditions, such as autoimmune disease, asthma, allergy, and eczema; no neurological conditions, such as epilepsy; no suspected vision, hearing or walking problems; no other health problems, such as cardiovascular, lung, and kidney diseases; and not taking any medications and no recent infections or vaccinations at the time of study enrollment. No family history of ASD was another eligibility criterion for the control subjects. In addition, control subjects were screened using the Social Communication Questionnaire (lifetime version) with a cutoff score <12 for eligibility to rule out the risk of ASD in our control subjects. Control subjects were frequency-matched to children with ASD based on age, sex, and nationality.

All participants with ASD had a confirmed clinical diagnosis of ASD by qualified professionals according to the Statistical Manual of Mental Disorders (DSM-5) and Autism Diagnostic Observation Schedule second edition (ADOS-2). Subjects with ADOS-2 scores < 7 were considered to have mild-to-moderate ASD, while subjects with ADOS-2 scores ≥ 7 were considered to have severe ASD.

### 4.2. Plasma Isolation and Processing

Whole blood samples were collected from the study participants into EDTA-containing anticoagulant tubes at HMC or PHCC sites and processed in the research facility at Qatar Biomedical Research Institute (QBRI) within two hours of sample collection. According to the manufacturer’s protocol, the plasma was isolated using density gradient centrifugation with Histopaque-1077 (Cat. #10771, Sigma-Aldrich, Saint Louis, MO, USA). Plasma was collected from the upper layer into a new tube, centrifuged at ~1800× *g* for 15 min, then aliquoted into 200 µL per tube and stored at –80 °C until further analysis.

### 4.3. Total RNA Extraction from Plasma

Frozen plasma samples were thawed in a water bath at 37 °C. As recommended by the manufacturer, 200 µL of cell-free (cf) plasma was pipetted into new tubes for RNA extraction using the miRNeasy Serum/Plasma Advanced Kit (Cat. #217204, Qiagen, Hilden, Germany). Due to the scarcity of cfRNA, the procedure was modified using 400 μL of plasma to extract total RNA. Each sample also contained 52 Qiaseq miRNA Library QC Spike-ins (Cat. #331541, Qiagen, Germany) that served as an internal control for miRNA expression analysis in plasma. This study adhered to the MIQE (i.e., Minimum Information for Publication of Quantitative Real-Time PCR Experiments) guidelines for miRNA-based research.

### 4.4. Quality Check of the Extracted RNA

The QIAseq miRNA Library QC qPCR Assay Kit (Cat. #331551, Qiagen Inc., Valencia, CA, USA) was used to determine the quality of the RNA before preparing the library for small RNA sequencing and to assess data quality post-Next Generation Sequencing (NGS). This kit contains 52 Spike-In controls and a qPCR panel to assess the technical noise across the whole workflow of the experiment, from RNA extraction to PCR and sequencing (to assess read reproducibility). This also helps detect enzymatic inhibitors, nucleases, and hemolysis, which are crucial in identifying plasma miRNAs. The procedure started with the usage of 52 QIAseq miRNA Library QC Spike-Ins during the RNA extraction step. Sample evaluation was performed via qRT-PCR. The RNA isolation efficiency was determined by calculating the delta Ct for UniSp100 (CT range). The results showed that the UniSp101 (Ct range: 31–34) and UniSp101 (Ct range: 25–28) levels fell within the general guidelines of approximately 5–7. To assess the effects of inhibitors, the UniSp6 value was calculated, and it was <2 Ct between any two samples. For hemolysis, the delta Ct (miR-23a–miR-451a) was no more than 5. A delta Ct value of 5–7 was considered mild, whereas samples with delta Ct values higher than seven were excluded from the study.

### 4.5. Small RNA Library Preparation

To construct the library and for molecular indexing, the QIAseq miRNA Library Kit (Cat. # 331505, Qiagen, Germany) and the QIAseq miRNA NGS 96 Index IL (Cat. #331565, Qiagen, Germany) were used. The most common method of transforming circulating miRNAs to a standardized level involves the use of similar amounts of biofluids and total RNA isolated, with the help of spike-ins as reference controls. Five microliters of total RNA column eluate were used for library preparation. The RNA samples were then ligated with 3′ and 5′ adapters, and the resulting samples were reverse transcribed into cDNA constructs. This reverse transcription step involves selecting RNA fragments with adaptors attached at both ends. The reverse transcription primer, which incorporates a Unique Molecular Indices (UMI) tag, was hybridized to the target miRNA and the 3′ adapter. Then, the miRNAs ligated at the 3′ and 5′ ends were reverse transcribed while receiving a UMI label. A universal sequence was incorporated during the reverse transcription. The sample indexing primers identified this sequence when the library was amplified. The cDNA constructs were extracted using a magnetic bead-based method, which was performed with slight modifications. The libraries were then amplified without selectivity using a dried universal forward primer from a plate and one of the 96 dried reverse primers from the same plate (Cat. #331565, Qiagen, Germany). This process ensured that each sample was given a unique custom index. After library amplification, a cleanup was performed using the same magnetic bead-based method. A library quality test was performed using the Agilent Technologies 2100 Bioanalyzer with the Agilent High Sensitivity DNA assay (Cat. #G2938-90020, Agilent Technologies, Santa Clara, CA, USA) and a peak at around 141 bp was observed in the purified library.

### 4.6. High-Throughput Sequencing of Small RNAs

To quantify the cDNA libraries, a Qubit Fluorometer and a Qubit HS dsDNA Assay Kit (Cat. #Q32854, Thermo Fisher Scientific, Waltham, MA, USA) were used, and the concentration was calculated from the average size obtained by the bioanalyzer. For sequencing, the libraries were diluted to a final concentration of 10 nM in a resuspension buffer and then indexed and multiplexed for sequencing on the Illumina platform. The final concentration for loading was determined to be 3 nM, and clustering was performed on the cBot2. Paired-end sequencing was performed on the Illumina HiSeq 3000/4000 SBS Kit with 150 cycles. The aim was to obtain up to 20 million reads per sample to identify new miRNAs. The adapters were cut from the sequences; the raw data from the Illumina HiSeq 3000/4000 platform were transformed from bcl2 format to fastq format.

### 4.7. Read Mapping and Annotation of Small RNA Sequences

The raw sequence files in bcl format obtained via the Illumina HiSeq 3000/4000 platform were then converted to fastq format using bcl2fastq v1. 8. 4 software (Illumina, Hayward, CA, USA). After this conversion, the reads were filtered, and the adapters were trimmed from them. The quality of the trimmed reads was then checked with the help of FASTQC; which allowed the removal of low-quality reads.

### 4.8. Quality Check of Sequencing Data and Abundance of miRNAs Within the Sample Cohort

The QIAseq miRNA sequencing data were analyzed using the CLC Genomics Workbench software to ensure that the sequencing data had a reliable distribution. The reads were processed as follows. For each sample, 20–30 million reads were obtained, more than 90% of the reads were mapped to the human genome (hg19), and approximately 70% of these sequences were considered small RNAs (sRNAs), representing sequences between 18 and 43 nt. The Biomedical Genomics Analysis plugin in the CLC Genomics Workbench software was used to quantify the expression of each miRNA sample that was annotated and submitted to miRBase. Approximately 2632 different human miRNA sequences were found in the samples, accounting for ~1 × 10^6^ to 10 × 10^6^ reads per each sample.

### 4.9. Bioinformatics Data Analysis

As previously described [18,34], the collected data were subjected to analysis, and the FASTQ sequence files were aligned to the human reference genome assembly version hg38 via the CLC Genomics Workbench software (version 24), allowing for one mismatch and a minimum read length of 18 nucleotides. Further analysis was performed with CLC Genomics Workbench using small RNA tools, and the spike-in reads were excluded. The “perfect match” was employed for spike-in reads during the dataset’s mapping, filtering, and counting. These spike-in reads were then expressed as a percentage of total reads per sample, and correlation plots were generated for all pairwise sample comparisons. R squared values of 0.95 and 0.99 were anticipated. Any sample falling outside these ranges was considered a potential technical outlier, and the data could be removed. The Qiagen miRNA Quantification workflow was applied to each sample using the Biomedical Genomics Analysis plugin, which is compatible with QIAseq miRNA Library Kit sequencing. The samples were first aligned to the miRBase database version 22 (http://www.mirbase.org/ accessed on 11 May 2024). The QIAseq miRNA Quantification tool also provides grouping of miRNAs based on the miRNA type as mature miRNA or by the miRNA seed sequence, making it possible to have the same mature miRNA from different precursors or the same seed sequence in different mature miRNAs.

### 4.10. Differential Expression Analysis

The workflow uses multi-factorial statistics with a negative binomial Generalized Linear Model (GLM) to determine differential expressions. The Mature and Seed transcriptome annotation table and the Grouped on Mature and Grouped on Seed expression table were used. Introducing the Integrated Unique Molecular Indices provided a way to count the absolute number of miRNA molecules, thereby overcoming PCR and sequencing bias. The miRNAs were considered to have significant differences if they had more than 50 counts of reads with a cutoff absolute fold change of 1.5 and an adjusted FDR *p*-value below 0.05 [35,36].

### 4.11. Advanced miRNA Pathway and Network Analysis

To explore the potential functional roles of these miRNAs and to perform pathway analysis and molecular network comparisons, we employed the Ingenuity Pathway Analysis (IPA) software version 127006219 (Qiagen Inc., CA, USA). This is because IPA provides a comprehensive list of pathway information. The data are extensive and suitable for pathway crosstalk analysis since almost all the molecules and their interactions are included. The IPA system utilizes Fisher’s exact test to determine if the pathways observed are significantly associated with the miRNAs of interest. Moreover, the IPA network analysis can pinpoint the molecular pathways by integrating information from a commercial database, encompassing information from the literature, gene expression profiles, and gene annotations.

### 4.12. BaseSpace Correlation Engine

BaseSpace Correlation Engine (BSCE; Illumina) was used to investigate the target genes of the differentially expressed miRNAs in independent studies conducted on the blood and brain tissues of individuals with ASD. The BSCE platform utilizes data deposited in international public repositories and employs powerful data-driven correlation, aggregation, and machine learning applications to process omics-scale data through standard, platform-specific pipelines. BSCE contains Bioset lists of highly curated, quality-controlled, and statistically significant genes, with each gene from RNA studies passing a cutoff absolute fold change of 1.2 and a *p*-value < 0.05. This platform runs an Illumina-developed Running Fisher algorithm that compares an imported query bioset with a target bioset, analogous to the Gene Set Enrichment Analysis (GSEA) method [37,38]. BSCE uses rank-based statistics to compute bioset–bioset correlations between the imported data and BSCE-curated studies. Overall, BSCE allows researchers to place their experimental results within the rich context of expert-curated omics experiments to discover novel associations, design new experiments, and validate their results with independent observations.

### 4.13. Statistical Analysis

Data were analyzed using the chi-square test for categorical variables and the Mann–Whitney U test for continuous variables. Correlation analysis was conducted using Spearman’s rho method for continuous variables. Demographic data were analyzed with SPSS (version 26.0), while correlation data were analyzed using GraphPad Prism (version 10). For differential gene expression analysis, data were considered statistically significant when the adjusted FDR *p*-value was below 0.05, with an absolute cutoff fold change of 1.5.

For the binary logistic regression, data were log2-transformed prior to the analysis. The outcome of interest was the diagnostic group, either ASD or control. In the initial model, we included all significantly altered markers as predictors and adjusted for child age and sex as covariates. A 10% change in the levels of the β-coefficient of each predictor was used to determine which variables to retain in the final model. Adjusted odds ratios (ORs) and 95% confidence intervals (CIs) were calculated to measure the association between each marker and ASD diagnosis relative to controls. Receiver operating characteristic (ROC) curves were constructed for the logistic regression model to evaluate the diagnostic accuracy of the selected markers in distinguishing between ASD and control cases. The area under the curve (ROC-AUC) was computed for the combination of selected markers using a nonparametric method. Data were analyzed using SPSS (version 26.0), and statistical significance was determined when the *p*-value was below 0.05.

## Figures and Tables

**Table 1:** Demographic characteristics of the study population.

	Total (n = 108)	Control (n = 42)	ASD (n = 66)	*p*-Value
Sex				
Male Female	87 (80.6) 21 (19.4)	33 (78.6) 9 (21.4)	54 (81.8) 12 (18.2)	0.678
Age in years	3.44 (2.99–3.75)	3.37 (2.93–3.81)	3.46 (3.11–3.75)	0.504
Family history of ASD				
Yes No	– –	NA NA	13 (19.7) 53 (80.3)	–
Consanguinity				
Yes No	33 (30.6) 75 (69.4)	15 (35.7) 27 (64.3)	18 (27.3) 48 (72.7)	0.353
Method of reproduction				
Natural Assisted (IVF)	103 (95.4) 5 (4.6)	41 (97.6) 1 (2.4)	62 (93.9) 4 (6.1)	0.647
Maternal complications				
Yes Diabetes Asthma Allergy Hypertension	40 (37) 23 (21.3) 2 (1.9) 3 (2.8) 3 (2.8)	15 (35.7) 11 (26.2) 1 (2.4) 0 (0) 0 (0)	25(37.9) 12 (18.2) 1 (1.5) 3 (4.5) 3 (4.5)	0.820
Multiple conditions (asthma, allergy, diabetes, and hypertension)	9 (8.3)	3 (7.1)	6 (9.1)	
No	68 (63)	27 (64.3)	41 (62.1)	
Pregnancy duration				
<9 months ≥9 months	6 (5.6) 102 (94.4)	1 (2.4) 41 (97.6)	5 (7.6) 61 (92.4)	0.401
Maternal age at labor				0.570
Age in years <35 years ≥35 years	29 (26.00–34.00) 87 (80.6) 21 (19.4)	30.00 (27.00–33.00) 36 (85.7) 6 (14.3)	29.00 (26.00–34.00) 51 (77.3) 15 (22.7)	0.280
Type of delivery				
Normal C-section Induced	65 (60.2) 39 (36.1) 4 (3.7)	27 (64.3) 15 (35.7) 0 (0)	38 (57.6) 24 (36.74) 4 (6.1)	0.254
Postnatal complications				
Yes Hypoxia Jaundice Hypoxia and Jaundice Others No	11 (10.2) 4 (3.7) 4 (3.7) 1 (0.9) 2 (1.9) 97 (89.8)	2 (4.8) 1 (2.4) 0 (0) 0 (0) 1 (2.4) 40 (95.2)	9 (13.6) 3 (4.5) 4 (6.1) 1 (1.5) 1 (1.5) 57 (86.4)	0.094
Birth weight				
Weight in kg	3.01 (2.80–3.50)	3.00 (3.00–3.52)	3.03 (2.57–3.50)	
<2.5 kg ≥2.5 kg	12 (11.1) 96 (88.9)	2 (4.8) 40 (95.2)	10 (15.2) 56 (84.8)	0.262 0.094
Nationality				
Egyptian Qatari Syrian Yemeni Sudanese Jordanian Others	26 (24.1) 21 (19.4) 19 (17.6) 16 (14.8) 13(12.0) 2 (1.9) 11 (10.2)	12 (28.6) 5 (11.9) 11 (26.2) 4 (9.5) 7 (16.7) 1 (2.4) 2 (4.8)	14 (21.2) 16 (24.2) 8 (12.1) 12 (18.2) 6 (9.1) 1 (1.5) 9 (13.6)	0.121

Data are presented as medians (lower–upper quartile) or n (%). *p*-values were assessed using the Mann–Whitney U test for continuous variables and Pearson’s chi-square or Fisher’s exact test as appropriate for categorical variables.

**Table 2:** Dynamic changes in circulating miRNAs with the age of children with ASD.

**Cohort age 2–4 years**			
**Group**	**miRNA**	**r**	***p*-Value**
ASD	**hsa-miR-182-5p**	0.254	0.040
	**hsa-miR-183-5p**	0.246	0.046
	**hsa-miR-335-5p**	−0.250	0.043
	**hsa-let-7b-5p**	0.244	0.048
	hsa-miR-92b-3p	0.270	0.028
	hsa-miR-486-5p	0.243	0.049
	hsa-miR-146a-5p	−0.245	0.047
	hsa-miR-532-5p	0.256	0.038
	hsa-miR-10b-5p	0.318	0.009
Control	hsa-miR-92b-3p	−0.352	0.022
	hsa-miR-365a-3p	0.476	0.025
	hsa-miR-9-5p	0.413	0.009
**Cohort age 5–12 years**			
**Group**	**miRNA**	**r**	***p*-value**
ASD	**hsa-miR-182-5p**	−0.355	0.005
	**hsa-miR-183-5p**	−0.341	0.008
	**hsa-miR-335-5p**	0.311	0.016
	**hsa-let-7b-5p**	−0.413	0.001
	**hsa-miR-7-5p**	−0.331	0.010
	**hsa-miR-4742-3p**	−0.367	0.007
	hsa-miR-141-3p	−0.369	0.004
	hsa-miR-196a-5p	−0.332	0.012
	hsa-miR-148b-5p	−0.412	0.003
	hsa-miR-328-3p	0.329	0.010
	hsa-miR-224-5p	0.260	0.045
	hsa-miR-744-5p	0.276	0.033
	hsa-miR-221-3p	0.396	0.002
Control	**hsa-miR-182-5p**	−0.857	0.011

N.B. miRNAs in bold are within the 17-miRNA signature shared between the two cohorts.

**Table 3:** List of 17 validated miRNA signatures exhibiting differential expression in ASD and their potential implications in ASD.

Gene Name	Fold Change	Adjusted *p*-Value	Role in Disease	Implications in ASD	Reference
miR-335-5p	2.945	1.993 × 10^−17^	Brain development, neural differentiation, migration; involved in cancer metastasis.	Contributes to abnormal brain development in ASD.	[19]
miR-3613-5p	2.962	1.194 × 10^−9^	Involved in gene regulation; less studied.	Role in ASD unclear.	[20]
miR-182-5p	−2.171	1.194 × 10^−9^	Sensory processing, neural development; implicated in cancer.	Relevant to sensory processing abnormalities in ASD.	[12]
miR-19a-3p	2.121	2.631 × 10^−8^	Part of miR-17-92 cluster; involved in cancer and immune regulation, cell proliferation, apoptosis.	Relevant to immune regulation and apoptosis in ASD.	[19]
miR-1180-3p	−2.508	5.482 × 10^−8^	Linked to cancer; involved in cell cycle regulation, apoptosis.	Relevant to cell cycle regulation and apoptosis in ASD.	[21]
miR-375-3p	−2.041	9.618 × 10^−6^	Pancreatic function, insulin secretion; implicated in various cancers.	Role in ASD unclear.	[8]
miR-15a-5p	1.631	2.703 × 10^−5^	Apoptosis, cell cycle regulation (cancer, cardiovascular diseases).	Relevant to neuronal death and abnormal cell cycles in ASD.	[20]
miR-7-5p	−1.560	1.247 × 10^−4^	Neuroprotection, synaptic plasticity; implicated in Parkinson’s disease, insulin signaling.	Influences synaptic and neurodevelopmental abnormalities in ASD.	[22]
let-7b-5p	−1.620	2.439 × 10^−4^	Regulates cell differentiation, maintains stem cell populations; involved in cancers and development.	Regulates developmental timing, cellular differentiation; potentially contributing to ASD.	[14]
miR-183-5p	−1.717	5.641 × 10^−4^	Sensory organ development, neuronal function, plasticity; implicated in various cancers.	Important for sensory processing and neural connectivity in ASD.	[10]
miR-4742-3p	−2.111	1.487 × 10^−3^	Less characterized; possibly involved in gene regulation.	Role in ASD unclear.	[13]
miR-12136	1.850	1.863 × 10^−3^	Linked to gene expression regulation; less studied.	Role in ASD unclear.	[21]
miR-199b-5p	−2.376	2.457 × 10^−3^	Cardiac and cancer biology; regulates HIF-1α, mTOR pathways; involved in neural plasticity.	Implicated in neurodevelopmental abnormalities.	[22]
miR-99a-5p	−1.635	3.233 × 10^−3^	Involved in cancers (prostate, breast); regulates cell proliferation and apoptosis.	May regulate immune responses and cell differentiation.	[8]
miR-1301-3p	1.502	2.118 × 10^−2^	Implicated in cancer, regulates cell proliferation and migration.	Could be linked to neurodevelopmental processes disrupted in ASD.	[10]
miR-4433b-5p	1.516	2.423 × 10^−2^	Gene expression regulation; less studied in neurodevelopment.	Potential role in gene expression modulation in the brain.	[11]
miR-127-3p	−1.508	2.428 × 10^−2^	Regulates cell proliferation and differentiation, studied in cancer and liver fibrosis.	Potential regulatory role in neurodevelopment.	[12]

## Data Availability

The data that support the findings of this study are available from the corresponding author upon reasonable request.

## Supplementary Materials

The following supporting information can be downloaded at: https://www.mdpi.com/article/10.3390/ijms26052044/s1.
