# Correction: Salloum-Asfar et al. MicroRNA Profiling Identifies Age-Associated MicroRNAs and Potential Biomarkers for Early Diagnosis of Autism. *Int. J. Mol. Sci.* 2025, *26*, 2044

**DOI:** 10.3390/ijms262311640

**Published:** 2025-12-01

**Authors:** Salam Salloum-Asfar, Samia M. Ltaief, Rowaida Z. Taha, Wared Nour-Eldine, Sara A. Abdulla, Abeer R. Al-Shammari

**Affiliations:** 1 Neurological Disorders Research Center, Qatar Biomedical Research Institute, Hamad Bin Khalifa University, Qatar Foundation, Doha P.O. Box 34110, Qatar; ssalloumasfar@hbku.edu.qa (S.S.-A.); sltaief@hbku.edu.qa (S.M.L.); rotaha@hbku.edu.qa (R.Z.T.); wnoureldine@hbku.edu.qa (W.N.-E.); 2 College of Health and Life Sciences, Hamad Bin Khalifa University, Qatar Foundation, Doha P.O. Box 34110, Qatar

The authors wish to make the following corrections to this paper [1]. In the original publication, Table S1 was mistakenly left out of the Supplementary Materials folder during the final production steps. Table S1 needed to be added to the Supplementary Materials. The authors state that the scientific conclusions are unaffected. This correction was approved by the Academic Editor. The original publication has also been updated.
