# Supplementary material for: MicroRNA Profiling Identifies Age-Associated MicroRNAs and Potential Biomarkers for Early Diagnosis of Autism
Source: Int J Mol Sci. 2025 Feb 26;26(5):2044. doi: 10.3390/ijms26052044 (PMC11900285; doi:10.3390/ijms26052044)
Supplement: Supplementary file 1 [file ijms-26-02044-s001.zip › Figure S1.pdf]

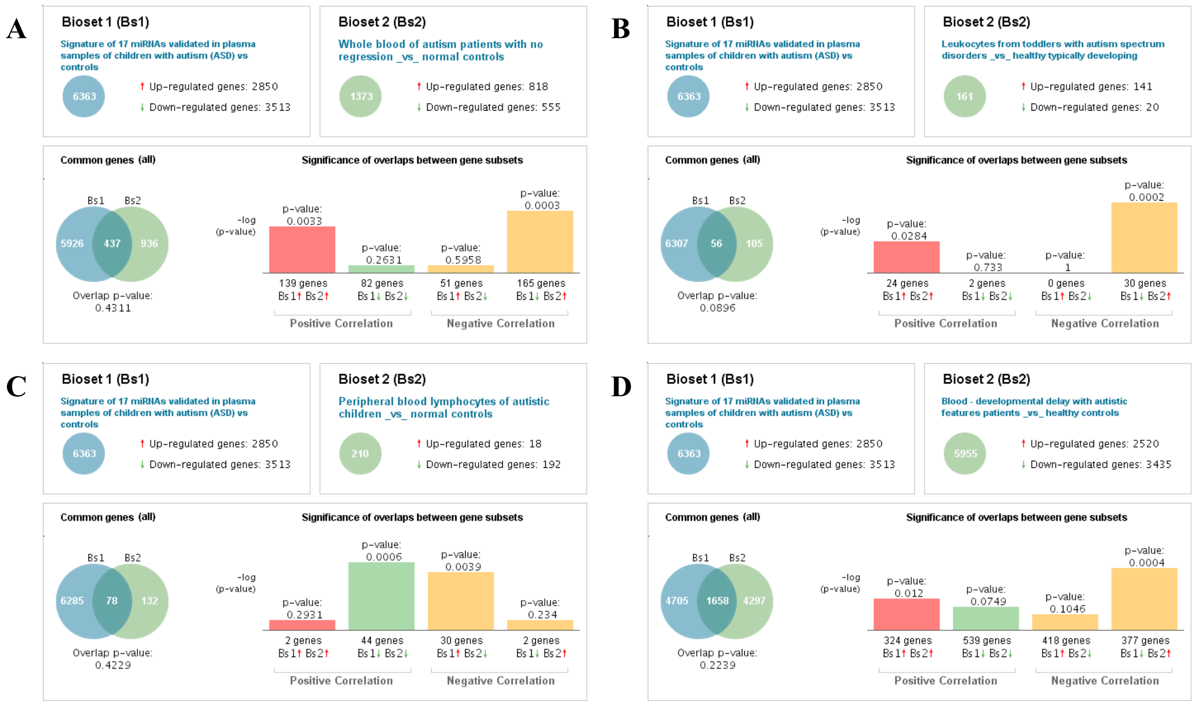

Figure S1. BaseSpace Correlation Engine (BSCE) analysis reveals dysregulated expression of the target genes of the 17-miRNA signature in human blood samples from individuals with ASD. (A) Correlation analysis shows 437 common target genes common between our dataset (Bioset 1) and independent study conducted on whole blood samples of subjects with ASD in comparison to their controls (Bioset 2). **(B)** Fifty-six common markers between our dataset (bioset 1) and independent study on leukocytes of toddlers with ASD vs. controls. **(C)** A total of 78 target genes are shared between our dataset (Bioset 1) and independent study on lymphocytes of subjects with ASD vs. the control group (Bioset 2). **(D)** A total of 1658 target genes are common between our dataset (Bioset 1) and independent study on blood samples of individuals with ASD compared to the control group (Bioset 2).
