# Supplementary material for: MicroRNA Profiling Identifies Age-Associated MicroRNAs and Potential Biomarkers for Early Diagnosis of Autism
Source: Int J Mol Sci. 2025 Feb 26;26(5):2044. doi: 10.3390/ijms26052044 (PMC11900285; doi:10.3390/ijms26052044)
Supplement: Supplementary file 1 [file ijms-26-02044-s001.zip › Figure S2.pdf]

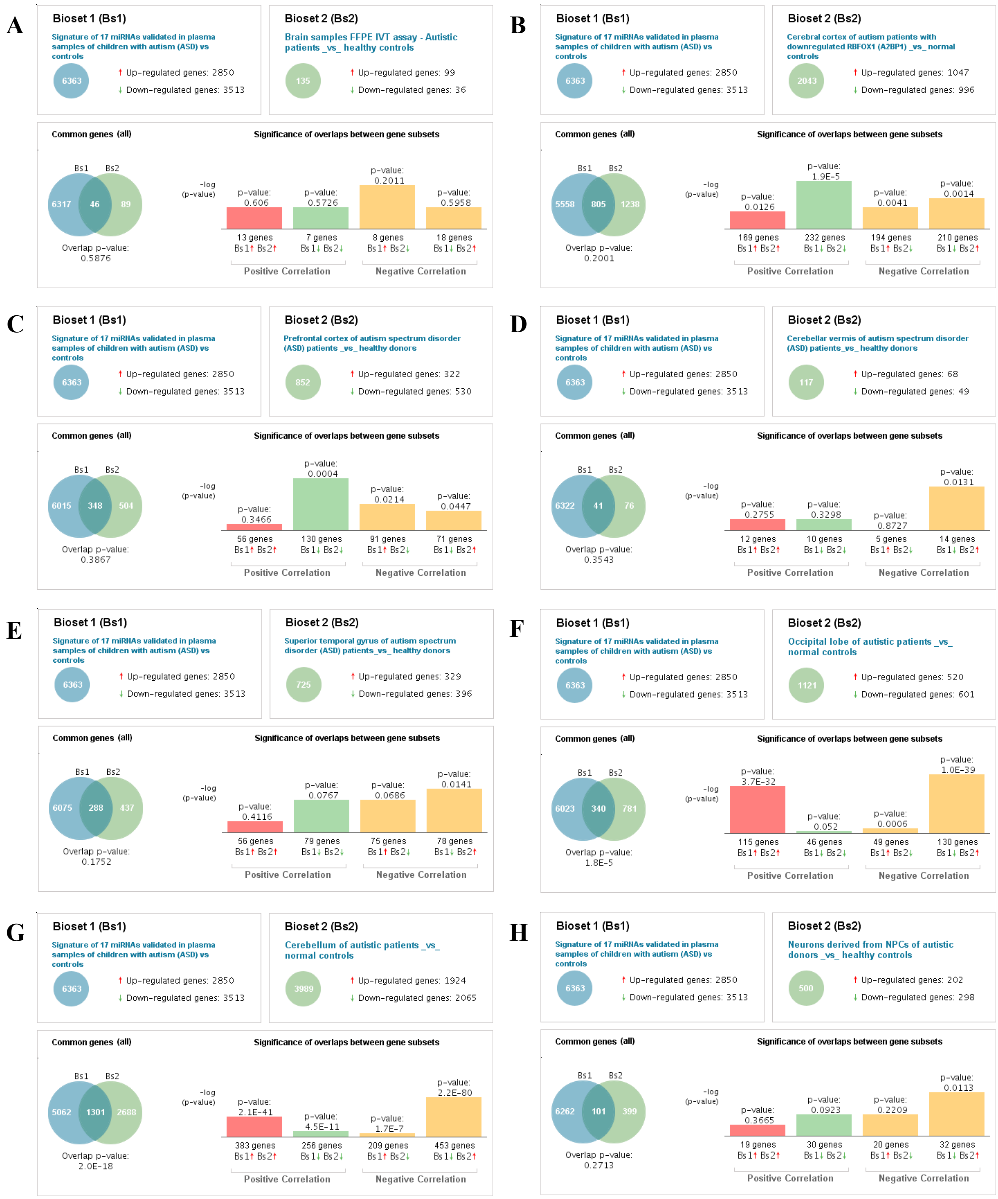

Figure S2. BSCE analysis shows dysregulated expression of the target genes of the 17-miRNA signature in human brain samples from individuals with ASD. (A) Correlation analysis reveals 46 common target genes between our dataset (Bioset 1) and independent study conducted on brain samples of subjects with ASD vs. control group (Bioset 2). **(B–C)** A total of 805 and 348 target genes are common between our dataset (Bioset 1) and other studies on cerebral cortex and prefrontal cortex of individuals with ASD vs. controls, respectively (Bioset 2). **(D–G)** A total of 41, 288, 340, and 1301 targets genes are shared between our dataset (Bioset 1) and independent studies on cerebellar vermis, superior temporal gyrus, occipital lobe, and cerebellum of subjects with ASD vs. the control group, respectively (Bioset 2). **(H)** A total of 101 target genes are common between our dataset (Bioset 1) and another study on neurons derived from individuals with ASD vs. controls (Bioset 2).
